# Identification of potent antibacterial inhibitors targeting methyltransferase Mtr1/TrmD in *Haemophilus influenzae* via molecular dynamics simulations

**DOI:** 10.1371/journal.pone.0328497

**Published:** 2025-08-28

**Authors:** Saud Almawash, Sitah Alharthi

**Affiliations:** 1 Department of Pharmaceutics, College of Pharmacy, Shaqra University, Shaqra, Saudi Arabia; 2 Department of Pharmaceutics, College of Pharmacy, Shaqra University, Al-Dawadmi Campus, Al-Dawadmi, Saudi Arabia; Kafrelsheikh University Faculty of Pharmacy, EGYPT

## Abstract

Bacterial influenza is a significant global health and economic concern, and the effectiveness of current therapies is declining as bacterial resistance increases. This case emphasizes the need for novel therapeutic approaches. A target-based method was used in this study to investigate the RNA 2’-O-methyltransferase MTr1/TrmD, an important enzyme involved in the pathogenic bacteria’s cap-snatching mechanism. This post-translational modification is critical for bacterial pathogenicity, providing opportunities for the development of novel inhibitor compounds. Computational screening revealed numerous interesting small-molecule inhibitors that could efficiently limit MTr1 activity, resulting in antibacterial effects. Notably, Sinefungin, a recognized inhibitor, had a binding affinity of −7.2 kcal/mol, which was lower than the top three inhibitors tested: Molecule 45 (−8.7 kcal/mol), Molecule 55 (−8.5 kcal/mol), and Molecule 56 (−8.5 kcal/mol). Additional confirmation using molecular dynamics simulations indicated significant structural changes in the control-MTr1 complex, particularly at the transitions from loop to helix and helix to loop. The leading inhibitors, on the other hand, maintained stable connections with the active site residues throughout a 120 ns simulation. Binding free energy estimates (MM/PBSA and MM/GBSA), as well as water swap investigations, revealed that Molecule 56 had the highest binding affinity of the inhibitors studied. This is followed by waterswap analysis where the compound 56 remains the prominent one in terms of higher binding affinities. Hence, it has been found from computational studies that our inhibitors remain more static which will ease a way for experimentalists towards *in vitro* and *in vivo* studies. These findings indicate that the discovered compounds, particularly Molecule 56, have the potential for future in vitro and in vivo validation, paving the door for the development of novel antibacterial therapeutics against *Haemophilus influenzae*.

## 1 Introduction

Antimicrobial resistance is a major worldwide concern that must be addressed. Drug-resistant microorganisms are estimated to cause roughly 1.27 million fatalities per year [1]. Globally, respiratory infections brought on by bacterial and viral pathogens are becoming more and more concerning since they have a major influence on rates of morbidity and mortality [2]. *Haemophilus influenzae* stands out among the bacterial pathogens due to its role in a number of respiratory illnesses, such as bronchitis, otitis media, pneumonia, and more severe situations including meningitis and sepsis [3].

Given its significant significance in respiratory infections and the growing problem of resistant strains, *H. influenzae* was chosen as the study’s focus. Non-Hib serotypes and non-typeable *H. influenzae* (NTHi) strains have become more common in recent years, despite the fact that vaccines are available for some serotypes, such as *H. influenzae* serotype b (Hib) [4,5]. Even though vaccines have significantly reduced the prevalence of Hib-caused invasive illnesses, NTHi and other serotypes (a–f) are still common in the population and can cause both mild and serious infections. Current vaccines do not cover these non-Hib strains, particularly NTHi, which emphasizes the need for more study into alternative therapies. Given the growing antibiotic resistance observed in these strains, this scenario highlights the need to investigate alternate approaches to treating *H. influenzae* infections [6,7].

The inhibition of certain bacterial enzymes that are important for vital cellular functions is a promising approach to address the prevalent issue of *H. influenzae* infections. A key enzyme that helps methylate guanosine-37 (m1G37) in tRNA is methyltransferase Mtr1/TrmD, which is essential for altering bacterial RNA. This post-transcriptional alteration is necessary to ensure accurate protein synthesis, prevent frameshift mutations, and maintain translational precision. TrmD is a highly conserved enzyme present in several ESKAPE pathogens, including *H. influenzae*, which relies on it for survival [8]. Unlike some viruses, such as SARS-CoV-2, which can cap their own RNA molecules, *H. influenzae* acquires molecular caps for viral RNA production by a process known as cap snatching [9]. As a result, disrupting Mtr1 function prevents the production of molecular caps that could be transferred to viral RNA, so impeding viral replication. Mtr1/TrmD is a bacterial enzyme that is extensively conserved among prokaryotic species but not in humans, making it an appealing and selective target for the creation of antibacterial medications. Focusing on this enzyme is a viable way to combating *H.* influenzae infections, especially considering the growing problem of antibiotic resistance. By suppressing Mtr1/TrmD, bacterial translation is interrupted, reducing bacterial growth and survival. This feature makes it an ideal target for developing novel antimicrobial medicines to successfully manage *H. influenzae* infections [10].

In recent years, computational methodologies such as molecular docking and molecular dynamics simulations have emerged as indispensable instruments in the field of drug discovery [11–16]. *These in silico* platforms facilitate researchers in the exploration of extensive compound libraries, allowing for the estimation of binding affinities and the examination of interactions with target enzymes [17]. The goal of this work is to use these *in silico* techniques to find potent antibacterial drugs that target Mtr1/TrmD in *H. influenzae*. Mtr1/TrmD was selected as a target due to its critical role in bacterial protein synthesis and its promise as a universal pharmacological target for the treatment of *H. influenzae* infections, irrespective of the serotype. Potential inhibitors can be more precisely identified using these *in silico* techniques, and their antibacterial efficacy can then be further examined in lab conditions.

## 2 Methodology

**Fig 1:**
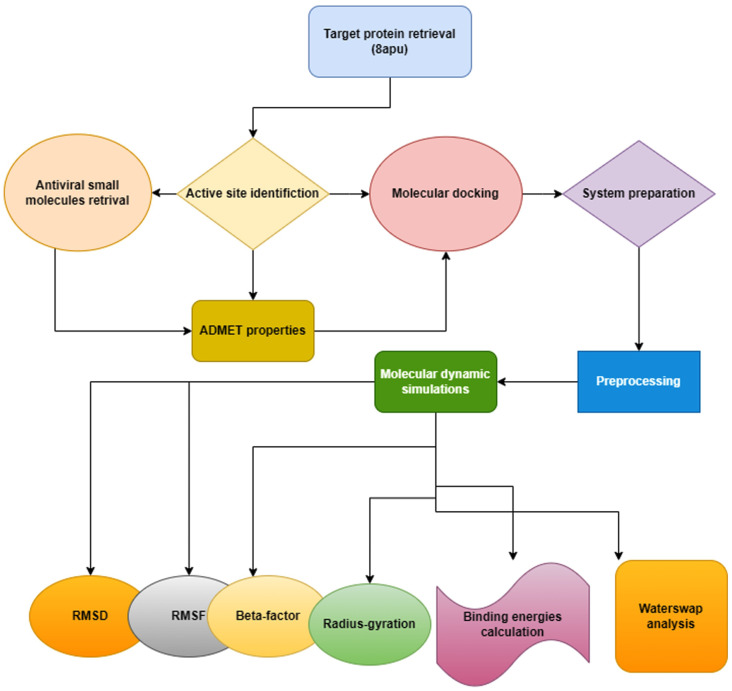
Flow chart of the current study.

This study employed *in-silico* techniques to identify potential inhibitors against the influenza virus protein target. The flow chart in **Fig 1** describes the entire methodology followed in this study.

### 2.1 Retrieval of antibacterial drugs and minimization

In this study, the antibacterial library https://www.chemdiv.com/catalog/focused-and-targeted-libraries/antibacterial-compounds-library/ was used for the retrieval of ligand and processing. The library was imported to https://www.inteligand.com/ligandscout/ [18], which employed the Lipinski rule of five to filter the compounds. Then, using an MM2 force field, the energy of the library was decreased. The reduced structures were then prepared for docking by being transformed into the pdbqt format. For docking PyRx [19] was applied where a minimized protein and ligands were uploaded.

### 2.2 Selection of target protein

The methyltransferase TrmD with pdb ID: 8apu retrieved from the protein data bank was subjected to minimization to improve the quality of the target by eradicating steric clashes [20]. The program used was UCSF CHIMERA [21], after assigning Gasteiger charges 1500 step of minimization were carried out with the aid of Tripos Force Field (TFF) [22]. After completing the sharpest descent steps, 750 steps of conjugate gradient are carried out to further enhance the structure. The steepest descent steps are utilized to eliminate extremely harsh steric conflicts. The minimization relaxes the structure through random search and systematic elimination [23] eventually, strengthens the bond geometry of the binding segments of the rigidly moving components [24].

### 2.3 Molecular docking

Molecular docking facilitates the determination of inhibitor-binding affinities and favorable binding conformations [25]. Antibacterial medications are docked using the freely available PyRx software [19]. To target the active domain of the methyltransferase TrmD receptor protein, we used a list of 5,000 filtered antibacterial chemical libraries together with a control inhibitor molecule. The docking is carried out by a standard genetic algorithm on an Intel Xeon QuadTM Core CPU running Linux. The XYZ coordinates of the grid box center were set to X = 45.0 Å, Y = 60.5 Å, Z = 38.2 Å, with dimensions of 30 × 30 × 30 Å to ensure full coverage of the binding pocket. The molecular docking parameters were applied precisely as specified for the single-docking experiment. The methyltransferase TrmD docking of molecules score with small inhibitor molecules was contrasted with sinefungin, the control inhibitor molecule. The complexes are subjected to further investigation using two programs: UCSF Chimera [21]. and Discovery Studio (DS) [26] and Visual Molecular Dynamic (VMD) [27]. It is important to carefully evaluate interaction alongside the binding value when selecting the best complexes.

### 2.4 Molecular dynamics simulation

To see how top docked complexes behave dynamically, molecular dynamics (MD) simulations were used. After simulating a situation and doing successive studies using its many components, the AMBER (Assisted model building with energy refinement) application is utilized [28]. The tleap program of AMBER16, it is used to build topology files and missing atoms were added. System solvation was performed with three-point convertible intermolecular potential (TIP3P) water, while, the force fields engaged for calculations include GAFF [22], and ff14SB [29]. The trajectories of MD simulations were examined through the AMBER PTRAJ module. These analyses are comprised of RMSD (Root Mean square Deviation), RMSF (Root Mean Square Fluctuation), β-factor, Rg (Radius of Gyration), Binding Energy (MM(PB/GB)SA) and waterswap analysis. For graphical analysis and evaluation of some of these parameters, a two-dimensional (2D) “Xmgrace” plotting tool is utilized [30].

### 2.5 Interaction analysis

The interaction analysis is performed with the help of DS. Interaction analysis plays a vital role in the analysis of any docked complex. It helps to detect the orientation and position of the ligand within the active site. Furthermore, it helps to define the stability of the complex. The interaction analysis includes both hydrogen and hydrophobic interactions.

### 2.6 MMPBSA and MMPGSA

MD simulations were subjected to MM(PB/GB)SA by AMBER16, to measure the complex’s binding free energy using trajectories [31]. They calculate free energies by forecasting the free energy difference between the ligand and protein alone and with complex. Both Poisson-Boltzmann (PB) and Generalized-Born (GB) techniques were used to carry out the analysis study individually.

### 2.7 Waterswap analysis

To compare the binding affinity of docked complexes, the Cresset Flare module employed the WaterSwap absolute binding free energy technique [32]. The WaterSwap energy calculation method is a Monte Carlo (MC) simulation method for calculating absolute protein-ligand binding free energies. WaterSwap started with the final confirmation of the MD simulations. Several approaches, including Bennett, thermodynamics integration (TI), and free energy perturbation (FEP), were utilized to calculate the binding free energy. Finally, by taking the arithmetic mean of the Bennett, TI, and FEP estimated energies, a consensus of binding free energy was reached. The investigation also discovered the hot spot amino acids that interacted with the ligand.

## 3 Results

### 3.1 Molecular docking

Molecular docking is a critical technique for predicting the primary binding mode of a ligand molecule to a biological macromolecule and assessing the binding affinity of the resulting complex. Traditionally, this process is known for its labor-intensive, time-consuming, and expensive nature. The advent of computer-aided drug design has undeniably made a substantial contribution to overcoming the many constraints of conventional drug design and streamlining the drug discovery process. Molecular docking rapidly found a vital role in modern structure-based drug design by enabling the prediction of the binding mode and the intermolecular framework of chemical interactions between small drug molecules and proteins that ultimately inhibit protein functionality. In this context, we focused on the top-selected compounds, which were directed toward the methyltransferase MTr1 TrmD receptor as shown in **Table 1**.

**Table 1:** Molecular docking results showing binding affinities of the small molecules along with control inhibitor (their 2D structures are given in S1 Fig).

S.no.	Chemical formula	Binding affinities
Compound-45	C19H25N5O3	−8.7 kcal/mol
Compound-56	C25H21FN4O32-	−8.5 kcal/mol
Compound-57	C26H24N4O32-	−8.5 kcal/mol
Compound-2	C12H15N3	−7.6 kcal/mol
Compound-5	C12H15N5	−7.4 kcal/mol
Compound-15	C8H9N5	−7.4 kcal/mol
Compound-20	C10H13N5	−7.3 kcal/mol
Compound-42	C11H14N4O	−7.3 kcal/mol
Compound-77	C23H24N4O32-	−7.2 kcal/mol
Control (Sinefungin)	C3H7NO5S	−7.2 kcal/mol

**Fig 2:**
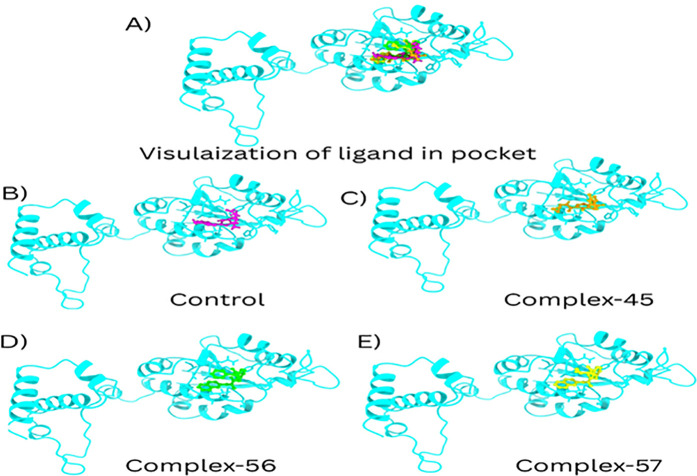
Structural visualization of ligand binding within the TrMD active pocket. **A**) Overview of the ligand-bound TrMD dimer showing positioning of all complexes. **B**) Control complex with the native ligand (magenta) showing partial pocket occupancy. **C**) Complex-45 (orange) stably situated within the binding site. **D**) Complex-56 (green) deeply embedded in the active pocket with close contact to key residues. **E**) Complex-57 (yellow) exhibiting deep insertion into the pocket, suggesting strong binding affinity.

These compounds were ranked based on their binding energy, leading to the identification of the three top molecules with the lowest binding energies, measured in kcal/mol. The most favorable binding complexes were as follows: TrmD-45 (−8.5 kcal/mol), TrmD-56 (−8.3 kcal/mol), and TrmD-57 (−7.8 kcal/mol). In comparison, the TrmD-control complex exhibited a binding score of −7.5 kcal/mol (**Fig 2**).

**Fig 3:**
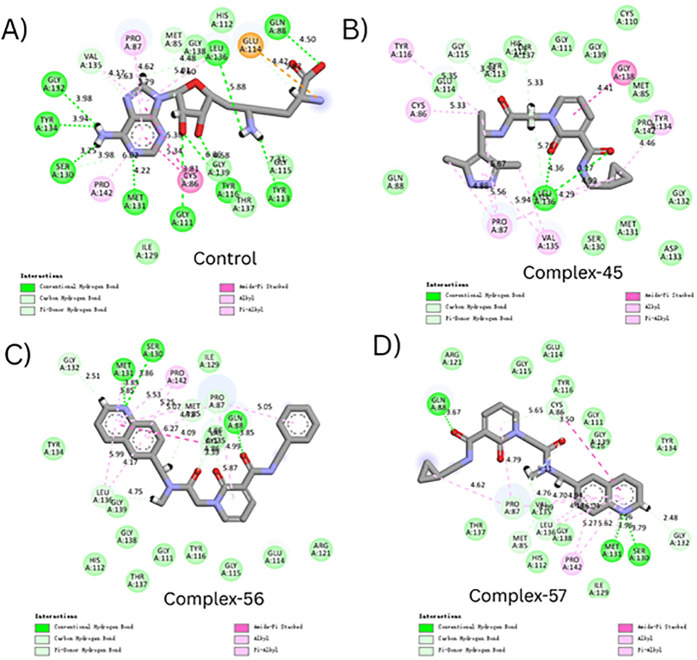
2D interaction diagram illustrating molecular interactions between TrMD and ligands. **A**) Control **B**) Complex-45 **C**) Complex-56 and **D**) Complex-57.

We conducted a thorough comparison between the co-crystallized (reference) ligand and the tested compounds (Complex-45, Complex-56, and Complex-57) within the TrmD protein’s active region in order to supplement the docking score analysis and obtain a deeper understanding of ligand–protein interactions. The 2D interaction diagrams (**Fig 3**) show that the control complex (Fig 3A) exhibited a clearly defined interaction network with both hydrophobic and polar contacts. The formation of conventional hydrogen bonds by GLN88 and GLU114 was noteworthy; their respective bond distances were 1.98 Å and 2.12 Å. Additionally, residues like CYS86, TYR116, MET131, LEU136, and PRO87 contributed π–alkyl, alkyl, and π–π interactions. By providing crucial stability inside the TrmD binding pocket—particularly with the involvement of TYR134, GLY111, and SER130—these non-covalent forces strengthened the ligand’s affinity.

An improved hydrogen bonding profile was seen in the TrmD–Compound-45 complex (**Fig 3B**), where significant polar contacts were formed by GLN88, TYR116, SER130, and TYR134. High-affinity binding was indicated by the fact that these hydrogen bonds were within the ideal ranges of 1.89 Å to 2.95 Å. Increased hydrophobic stability was further facilitated by π–alkyl interactions with residues including CYS86, PRO87, and LEU136. Compound-45 showed a more compact and extended binding profile than the control, indicating a stronger binding pocket interaction.

The TrmD–Compound-56 complex (**Fig 3C**), on the other hand, showed a comparatively small network of polar interactions. Only GLN88 and GLY132, whose bond lengths ranged from 2.40 to 2.74 Å, engaged in hydrogen bonding. Hydrophobic interactions, such as alkyl and π–alkyl contacts with PRO87, MET131, LEU136, and CYS86, were the main factor that stabilized the ligand. The decreased number of hydrogen bonds may result in a somewhat lower overall affinity as compared to Compound-45 and the control, even though the binding is maintained.

The interaction network of the TrmD–Compound-57 complex (**Fig 3D**) was evenly distributed and balanced. In addition to the π–alkyl and π–π interactions involving PRO87, MET131, and SER130, key residues like GLN88, CYS86, and LEU136 formed conventional hydrogen bonds (with distances ranging from 2.15 Å to 2.90 Å). Compound-57 offers both polar and hydrophobic stabilization within the active site, closely resembling the binding behavior of the reference ligand, as evidenced by the presence of several interaction types and appropriate bond lengths.

When combined, these results suggest that although all three compounds have significant interaction patterns inside the TrmD active site, Compound-45 and Compound-57 in particular have binding properties that are similar to the control. With important residues like GLN88, CYS86, MET131, SER130, and LEU136, they have the capacity to form numerous hydrogen bonds and hydrophobic interactions, indicating their great promise as lead candidates for additional experimental validation.

### 3.2 Molecular dynamic simulation

Molecular dynamics (MD) simulations were performed in an aqueous environment over a 100 ns timescale for all docked complexes (Complex-45, Complex-56, and Complex-57) as well as the co-crystallized (control) ligand in order to assess the structural stability and dynamic behavior of the TrmD-ligand complexes. To more accurately evaluate the relative stabilities and binding efficiencies of the novel compounds and the reference ligand, a direct comparison was made possible by this thorough approach.

**Fig 4:**
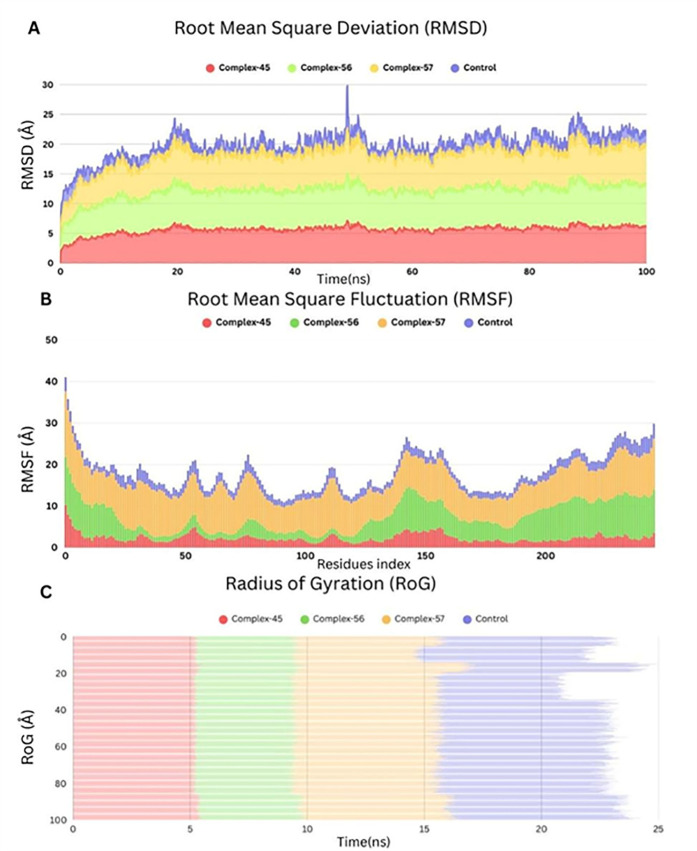
Comparative molecular dynamics analysis of trmd-ligands complexes. **A**) RMSD profiles showing structural stability over 100 ns **B**) RMSF indicating residue-level flexibility **C**) RoG depicting overall compactness during the first 25 ns.

The Root Mean Square Deviation (RMSD) of each complex’s backbone atoms is displayed in **Fig 4A**. With an average RMSD profile of roughly 1.65 Å, Complex-45 showed the least amount of structural deviation and the highest level of stability over time. Complex-57 displayed somewhat greater variability with an average of 1.75 Å, whereas Complex-56 trailed closely behind with an average RMSD of 1.62 Å. The control complex showed larger RMSD variations, with an average of 2.95 Å and occasional strong spikes up to ~31 Å. These differences suggest that the control complex has lower conformational stability than the newly bound molecules. The inclusion of the control complex in MD simulations was critical in supporting these findings by providing a baseline for stability comparison, indicating that the investigated compounds have greater structural retention within the active site under physiological settings.

Root Mean Square Fluctuation (RMSF) analysis (**Fig 4B**) corroborated these findings. The RMSF values, which measure residue-level flexibility, revealed that Complex-45 had the lowest average fluctuation (~1.35 Å), followed by Complex-56 (~1.3 Å) and Complex-57 (~1.4 Å). The control compound had higher variations among residues, especially in loop areas, reaching up to ~40 Å. These findings show that the test complexes have tighter residue-level binding and less flexibility, which increases their structural stability.

**Fig 4C** depicts the Radius of Gyration (RoG), a metric for determining the compactness of each complex. Complex-45 had the most compact structure, with an average RoG of 35.0 Å. Complex-56 and Complex-57 had comparable compactness, with average RoG values of 36.7 Å and 36.8 Å. The control complex had the least compact structure, with a RoG of 40.1 Å, indicating greater RMSD and RMSF measurements.

These MD simulation metrics (RMSD, RMSF, and RoG) together highlight the structural robustness and excellent dynamic behavior of the studied compounds, particularly Complexes 45 and 56. These complexes’ limited flexibility, high compactness, and low deviation suggest strong and stable interactions in the TrmD active site. The inclusion of the co-crystallized ligand as a reference proved the proposed compounds’ comparative advantage. These findings confirm their potential as lead candidates for future experimental validation and optimization.

**Fig 5:**
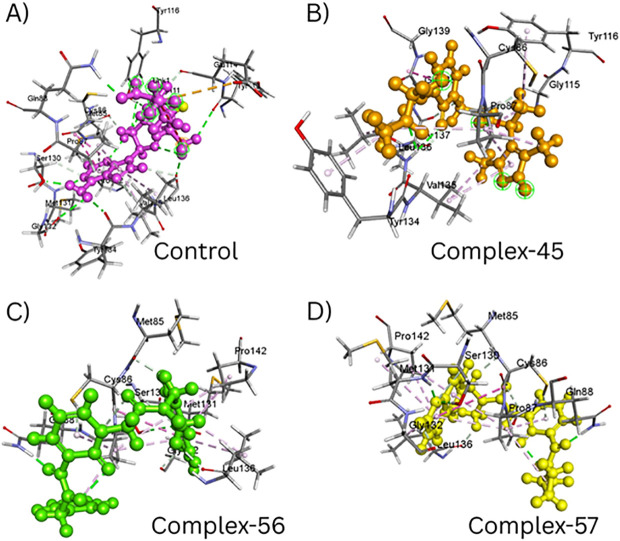
Depicting the hydrophobicity of all the complexes during the simulation time intervals.

To supplement the RMSD, RMSF, and RoG studies, the final structural conformations of the TrmD-ligand complexes obtained from molecular dynamics simulations were examined to analyze ligand placement, stability, and critical interactions (**Fig 5**). All systems displayed convergence during the simulation, demonstrating steady dynamics throughout the 100-ns trajectory.

Visual analysis revealed that the ligands in Complexes 45, 56, and 57 remained solidly anchored in the TrmD binding pocket with relatively slight conformational changes. These structural alterations appeared to improve the ligands’ interaction profile, primarily through stronger hydrophobic interactions and sustained hydrogen bonding. In contrast, the co-crystallized ligand (control complex) showed significant variations in binding orientation and a decrease in interaction density, indicating decreased stability under physiological settings. This structural shift supports the increased RMSD and RoG values found in the control complex.

Notably, Complex-56 and Complex-57 penetrated TrmD’s active site cavity the deepest, owing to an increase in hydrophobic interactions involving important residues such as Met131, Pro87, Leu136, and Cys86. These interactions were enhanced by stable hydrogen bonds created with residues such as Ser130, Gln88, and Gly132. The combination of hydrophobic embedding and polar contact stabilization emphasizes the improved binding efficiency of these two molecules.

The spatial arrangement of the ligands also confirms the RMSF data, with Complex-56 and −57 showing lower residue variations, indicating a more stiff and well-anchored complex. In contrast, the control complex exhibited greater flexibility and weaker anchoring, indicating a less effective interaction with the active site residues.

Collectively, these structural discoveries support the dynamic modeling data and confirm that Complex-56 and Complex-57, in particular, have strong, stable, and deep binding inside the TrmD active site. Their excellent hydrophobicity profiles and contact stability make them better options than both the control ligand and Complex-45. These findings support the continued prioritizing of Complexes 56 and 57 for lead optimization and experimental validation.

### 3.3 MMPB/GBSA energies calculations

To assess the stability of the complexes, we employed the MMGB/PBSA method to compute each system’s binding free energies, and the results are presented in **Table 2**. Notably, the TrmD-control complex exhibited relatively low binding energies, with values of −22.05 kcal/mol (MMGBSA) and −23.02 kcal/mol (MMPBSA). In contrast, the TrmD-45 complex displayed higher binding energies, with values of −39.72 kcal/mol (MMGBSA) and −41.09 kcal/mol (MMPBSA), while the TrmD-56 complex exhibited binding energies of −48.51 kcal/mol (MMGBSA) and −49.62 kcal/mol (MMPBSA), and the TrmD-57 complex had binding energies of −44.02 kcal/mol (MMGBSA) and −45.27 kcal/mol (MMPBSA). These findings suggest that the proposed ligand complexes have a high binding affinity. Furthermore, our analysis revealed that van der Waals and electrostatic interactions, in conjunction with non-polar solvent energy, contributed negatively to the overall interaction energy, while polar solvent energy made a positive contribution to the overall free binding energy. Across all complexes, van der Waals interactions made a more substantial negative contribution than electrostatic interactions in relation to the total binding energy. Non-polar free energy, relative to the total binding energy, contributed to a lesser extent. This implies that the selected complexes are primarily stabilized through non-polar solvation capacity, van der Waals, and electrostatic interactions. The findings from the MMGB/PBSA method further corroborate the docking results, confirming the stable behavior of the ligands.

**Table 2:** Binding energy values (kcal/mol) for filtered hits-TrmD complexes.

Parameter	Control	Complex-45	Complex-56	Complex-57
**MM-GBSA**				
Van der Waals Energy Term	−26.41	−41.30	−47.51	−44.00
Electrostatic Energy Term	−11.20	−18.09	−19.64	−16.34
Gas Phase Energy Term	− 37.61	− 59.39	− 67.15	−60.34
Solvation Energy Term	15.56	19.67	18.64	16.32
Net Energy Term	− 22.05	− 39.72	− 48.51	− 44.02
**MM-PBSA**				
Van der Waals Energy Term	−26.41	−41.30	−47.51	−44.00
Electrostatic Energy Term	−11.20	−18.09	−19.64	−16.34
Gas Phase Energy Term	− 37.61	− 59.39	− 67.15	−60.34
Solvation Energy Term	14.59	18.30	17.53	15.07
Net Energy Term	−23.02	−41.09	−49.62	−45.27

### 3.5 WaterSwap calculation

**Fig 6:**
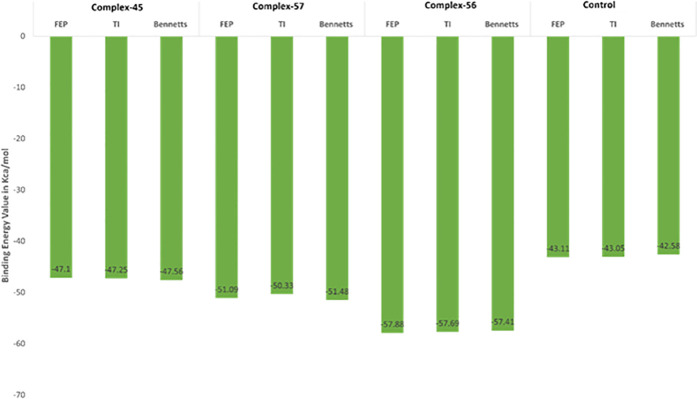
Binding free energy evaluation for complexes of compound-45, compound-56, compound-57, and control through WaterSwap calculations.

The WaterSwap function in the Sire package was utilized to assess the stability of the complexes and calculate the absolute binding free energy. This function leverages an explicit water model, allowing for a detailed examination of the interactions involving the protein, water, and ligand, as well as the protein-water and ligand-water interactions. This level of detail is crucial because when utilizing continuum solvent methods like MMGBSA, important interactions can be overlooked. In particular, water molecules play a vital role as they act as bridges in the interactions between the protein and the ligand. The accompanying **Fig 4** in our current study illustrates the calculation of free binding energy using WaterSwap, presenting the outcomes from three different algorithms for both ligands. The calculated binding affinity values with respect to the enzyme’s active pocket fall well within an acceptable range when compared to the established threshold of 1 kcal/mol. Notably, Compound-45, Compound-56, and Compound-57 exhibit stronger binding to the TrmD active site in comparison to the control ligand. The calculated findings suggest that compound 56 was the most prominent one in response to binding affinities with all the three parameters/algorithm showing FEP −57.88 kcal/mol, TI −57.69 kcal/mol and BENNETTS −57.41 kcal/mol followed by other complexes as sown in **Fig 6**.

### 3.4 Pharmacokinetic properties

A library of Antibacterial compounds underwent an evaluation based on physiochemical properties. The application of ADME (Absorption, Distribution, Metabolism, and Excretion) and the Lipinski rules served as valuable tools for researchers in the identification of the most promising druggable targets. It is noteworthy that all the compounds adhered to the Lipinski rule of five, indicating their potential suitability for drug development. Additionally, the calculation of the TPSA (Topological Polar Surface Area) parameter was important in assessing the membrane permeability of these compounds, and all of them demonstrated this desirable characteristic. Moreover, the inhibitors exhibited high water solubility and absorption properties. Notably, they were found to be non-inhibitors of the CYP2D6 enzyme and showed no signs of pan assay interference substructures. This collective set of attributes positions these inhibitors as ideal candidates for further experimental evaluation, considering their synthetic accessibility, as summarized in **Tables 3**, **4**. The results obtained from this comparative analysis provide valuable insights that will assist medicinal chemists in the design of compounds with enhanced biological potency. Subsequently, the top ten compounds identified through this analysis were further subjected to molecular docking studies.

**Table 3:** Lipinski Rule of five calculated for the selected compounds by SWISS-ADME.

Molecule	MW	Heavy atoms	Aromatic heavy atoms	Fraction Csp3	Rotatable bonds	H-bond acceptors	H-bond donors	TPSA
Control	169.16	10	0	0.67	3	6	3	126.07
Compound 2	169.16	10	0	0.67	3	6	3	126.07
Compound 5	201.27	15	0	0.5	1	2	2	38.36
Compound 15	229.28	17	10	0.42	1	4	2	76.72
Compound 20	175.19	13	0	0.5	1	4	2	63.08
Compound 42	203.24	15	9	0.4	1	3	3	79.62
Compound 44	218.26	16	9	0.45	1	4	1	51.97
Compound 45	371.43	27	0	0.58	9	4	3	104.61
Compound 56	444.46	33	6	0.24	8	5	1	81.85
Compound 57	440.49	33	6	0.27	9	4	1	81.85
Compound 77	404.46	30	0	0.43	9	4	1	81.85

**Table 4:** ADMET properties calculated for the selected compounds by SWISS-ADME.

Molecule	Water Solubility	GI absorption	BBB	CYP2D6 inhibitor	Bioavailability Score	PAINS
Control	Highly soluble	High	No	No	No	0
Compound 2	Very soluble	Low	No	No	No	0
Compound 5	Very soluble	High	No	No	No	0
Compound 15	Highly soluble	Low	No	No	No	0
Compound 20	Very soluble	High	No	No	No	0
Compound 42	Very soluble	High	No	No	No	0
Compound 44	Soluble	High	No	No	No	0
Compound 45	Soluble	High	No	No	No	0
Compound 56	Soluble	High	No	No	No	0
Molecule 57	Soluble	High	No	No	No	0
Compound 77	Highly soluble	High	No	No	No	0

## 4 Discussion

Bacterial influenza, an acute respiratory infection caused by the *H. influenzae*, presents a challenge due to the limited availability of effective treatments [33]. Due to the escalating problem of antibiotic resistance and the constraints of existing treatments for certain strains of *H. influenza,* considerable endeavors have been undertaken to identify novel antibacterial processes that may facilitate the creation of groundbreaking antimicrobial agents [34]. This research entailed a comprehensive examination of an antibacterial chemical library to identify possible inhibitors of Methyltransferase TrmD, a crucial enzyme in bacterial RNA modification. The selection method emphasized substances that met particular ADMET (Absorption, Distribution, Metabolism, Excretion, and Toxicity) criteria, guaranteeing acceptable pharmacokinetic features and safety profiles [35].

Out of initial set of 3500 compounds, the top ten were selected based on their compliance with the Lipinski rule of five [36]. These compounds demonstrated favorable characteristics, including the Topological Polar Surface Area (TPSA) parameter, which assessed their membrane permeability, revealing a desirable property of permeability [37]. Furthermore, these chosen inhibitors exhibited significant water solubility and effective absorption characteristics, while showing no inhibitory impact on the CYP2D6 enzyme. Moreover, the lack of pan-assay interference substructures (PAINS) validated their specificity as potential therapeutic candidates.

The selected top compounds, along with a control, were subjected to site-directed docking against the TrmD receptor. Sinefungin served as a control in this study due to its established role as a broad-spectrum methyltransferase inhibitor. Its structural resemblance to S-adenosylmethionine (SAM), the natural methyl donor utilized by TrmD in methylation reactions, enhances its relevance. Its capacity to competitively bind to the active site of methyltransferases, including TrmD, renders it an appropriate reference compound for evaluating the binding efficacy of newly identified inhibitors. By analyzing the binding interactions and stability of candidate compounds in comparison to Sinefungin, their relative effectiveness can be assessed in targeting TrmD. The application of Sinefungin in molecular docking and simulation studies sets a benchmark for evaluating the efficacy of novel inhibitors in disrupting the enzymatic activity of TrmD. Sinefungin serves as a crucial positive control, ensuring the reliability of both computational and experimental outcomes in this study, owing to its established inhibitory mechanism and structural similarity to TrmD [38].

Compound-45 (–8.5 kcal/mol), Compound-56 (–8.3 kcal/mol), and Compound-57 (–7.8 kcal/mol) were the three lead compounds that showed the highest binding affinities to the TrmD active site. According to thorough interaction investigations, the control complex formed strong hydrogen bonds with GLN88, GLU114, and TYR116, which were further reinforced by hydrophobic contacts with residues such as CYS86, PRO87, and MET131. Compound-45 had a more diverse interaction profile, including hydrogen bonds with GLN88, TYR116, TYR134, and SER130, as well as π-alkyl interactions with CYS86, PRO87, and LEU136. Compound-56 showed fewer polar interactions and relied more on hydrophobic contacts, indicating weaker binding. Meanwhile, Compound-57 closely resembled the control’s interaction pattern, combining hydrogen bonds with GLN88, CYS86, and LEU136 with hydrophobic contacts with MET131 and SER130, indicating stable and effective binding.

All complexes showed that four essential residues—LEU136, MET131, GLN88, and SER130—were involved in ligand anchoring. Since these residues are neutral or nonpolar, they emphasize the TrmD binding site’s predilection for ligands that can create non-covalent interactions, especially those that have hydrophobic or aromatic moieties. This implies that substances with these characteristics typically have stronger and more consistent binding inside the TrmD active pocket, which is an important requirement for the development of new inhibitors.

To further validate this hypothesis, the behavior of the compounds was closely monitored through approximately 100 ns of molecular dynamics simulations. The trajectory analysis confirmed a remarkably stable binding pattern for all systems in comparison to the TrmD-control complex. Furthermore, we conducted MMGB/PBSA calculations for all complexes, revealing significant differences in binding energies. Notably, the TrmD-control complex exhibited relatively high binding energies, whereas Compound-45, Compound-56, and Compound-57 demonstrated stronger interactions with the TrmD active site, further supporting their potential as positive inhibitors.

It is important to emphasize that computational validation alone is insufficient to prove the identified compounds’ efficacy, even though the *in silico* results clearly show that they have significant inhibitory potential against TrmD. Experimental investigations, including in vitro enzymatic assays, bacterial growth suppression assays, and in vivo infection models, are required to further evaluate the effectiveness of these drugs. The genuine inhibitory potential of these substances, their specificity for TrmD, and their general pharmacokinetic and pharmacodynamic characteristics can only be determined through laboratory-based validation. The medicinal promise of these substances is only speculative in the absence of this experimental validation. Future research should therefore focus on synthesizing these chemicals and thoroughly examining them biochemically and microbiologically to see how efficient they are as antibacterial agents against *H. influenzae*.

## 5 Conclusion

The present study addresses the critical challenge of combatting bacterial influenza, an acute respiratory infection caused by pathogenic bacteria. Given the limited availability of effective treatments, substantial efforts have been dedicated to identifying novel antibacterial mechanisms as a basis for the development of innovative anti-influenza drugs. Our research commenced with an extensive screening of an antibacterial compound library, guided by ADMET criteria. This was followed by molecular docking and in-depth molecular dynamics simulations. Among the screened compounds, Compound-45 (C₁₉H₂₅N₅O₃), Compound-56 (C₂₅H₂₁FN₄O₃), and Compound-57 (C₂₅H₂₁FN₄O₃) demonstrated strong inhibitory activity against Methyltransferase Mtr1/TrmD, an important enzyme involved in bacterial RNA modification and protein synthesis. Their interactions with key residues in the TrmD active site, as evidenced by computational investigations, emphasize their potential as effective inhibitors. Importantly, the structural and biochemical properties of Compounds 56 and 57, such as their fluorinated groups and nitrogen-rich frameworks, improve their binding affinity and stability, making them very intriguing candidates for future development. These findings provide useful insights into potential inhibitors, but further experimental validation is required using biochemical assays, bacterial growth inhibition investigations, and in vivo models to establish their efficacy and pharmacokinetic features. If successful, this validation could pave the door for new antibacterial medicines targeting *H. influenzae*, which is critical for closing a substantial gap in the treatment of respiratory infectious disorders.

## Supporting information

S1 Fig 2D structures of top small molecules and control that showed binding ability against methyltransferase MTr1 TrmD receptor. (TIF)
