# Supplementary figures and images for: Identification of potent antibacterial inhibitors targeting methyltransferase Mtr1/TrmD in Haemophilus influenzae via molecular dynamics simulations
Source: PLoS One. 2025 Aug 28;20(8):e0328497. doi: 10.1371/journal.pone.0328497 (PMC12393699; doi:10.1371/journal.pone.0328497)

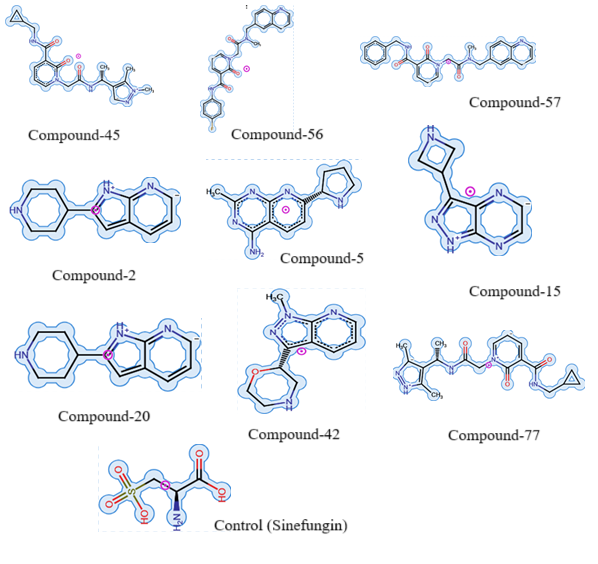

Supplement: S1 Fig — (TIF) [file pone.0328497.s001.tif]
